# Aberrantly Expressed OTX Homeobox Genes Deregulate B-Cell Differentiation in Hodgkin Lymphoma

**DOI:** 10.1371/journal.pone.0138416

**Published:** 2015-09-25

**Authors:** Stefan Nagel, Stefan Ehrentraut, Corinna Meyer, Maren Kaufmann, Hans G. Drexler, Roderick A. F. MacLeod

**Affiliations:** Department of Human and Animal Cell Lines, Leibniz-Institute DSMZ, German Collection of Microorganisms and Cell Cultures, Braunschweig, Germany; University of Navarra, Center for Applied Medical Research, SPAIN

## Abstract

In Hodgkin lymphoma (HL) we recently reported that deregulated homeobox gene MSX1 mediates repression of the B-cell specific transcription factor ZHX2. In this study we investigated regulation of MSX1 in this B-cell malignancy. Accordingly, we analyzed expression and function of OTX homeobox genes which activate MSX1 transcription during embryonal development in the neural plate border region. Our data demonstrate that OTX1 and OTX2 are aberrantly expressed in both HL patients and cell lines. Moreover, both OTX loci are targeted by genomic gains in overexpressing cell lines. Comparative expression profiling and subsequent pathway modulations in HL cell lines indicated that aberrantly enhanced FGF2-signalling activates the expression of OTX2. Downstream analyses of OTX2 demonstrated transcriptional activation of genes encoding transcription factors MSX1, FOXC1 and ZHX1. Interestingly, examination of the physiological expression profile of ZHX1 in normal hematopoietic cells revealed elevated levels in T-cells and reduced expression in B-cells, indicating a discriminatory role in lymphopoiesis. Furthermore, two OTX-negative HL cell lines overexpressed ZHX1 in correlation with genomic amplification of its locus at chromosomal band 8q24, supporting the oncogenic potential of this gene in HL. Taken together, our data demonstrate that deregulated homeobox genes MSX1 and OTX2 respectively impact transcriptional inhibition of (B-cell specific) ZHX2 and activation of (T-cell specific) ZHX1. Thus, we show how reactivation of a specific embryonal gene regulatory network promotes disturbed B-cell differentiation in HL.

## Introduction

In Hodgkin lymphoma (HL) infiltrated lymph nodes contain just a small number of the malignant Hodgkin/Reed-Sternberg (HRS) cells and many bystander cells, including activated lymphocytes, plasma cells and granulocytes [1]. This situation reflects aberrant expression of several signalling molecules comprising interleukins and other growth factors together with their receptors, resulting in constitutive activation of the associated pathway mediators including JAK-STAT, MAPK, and ERK1/2 [2,3]. Additionally, aberrant activities of NFkB transcription factors (TFs) promote survival of the HRS cells. Multiple mechanisms have been described which contribute to their activation in HL, including amplification of REL, and mutation of IkB and TNFAIP3/A20 [4].

Compromised B-cell development has been highlighted as a major aspect of the pathogenesis in HL from analysis of gene expression profiles of cell lines and microdissected primary HRS cells [5–7]. Main TFs important for B-cell development are absent or inactivated, resulting in B-cells with incomplete phenotypes [4]. Aberrantly downregulated B-cell TFs include PAX5, BOB1/OBF1, OCT2 and EBF1 [7–11]. Suppression of PAX5, BOB1 and OCT2 is responsible for the loss of immunoglobulin expression accompanying blocked B-cell development [10]. Furthermore, repression of TCF3/E2A activity by overexpressed ID2 and ABF1 proteins and ectopic activation of T-cell specific TF GATA3 are additional features of disturbed B-cell differentiation in HL [12–14]. However, reactivation of the basic TF PAX5 is alone insufficient to recover the B-cell program in HL, indicating that multiple factors are involved in coordinating B-cell differentiation [15].

HRS cells harbor multiple chromosomal aberrations which are, however, mostly non-recurrent, hampering identification of participant oncogenes [16–19]. Recently, a role for chromothripsis has been identified in HL cells manifested as non-directed focal genomic rearrangements whose oncogenomic role remains unclear [20,21]. Nevertheless, chromosomal and genomic alterations remain very likely to underpin malignant transformation in HL. Recently, we described a chromosomal aberration in HL cell line L-1236, t(4;8)(q27;q24), which involves the upstream regulatory region of the B-cell specific gene ZHX2 at 8q24, effecting its downregulation [22,23]. ZHX2 encodes a Zn-finger and homeodomain containing TF involved in the process of B-cell differentiation [24], further illustrating the oncogenic role of deregulated developmental factors in HL.

We have characterized more deregulated TFs involved in the pathogenesis of HL, including FOXC1 and MSX1 together with its repressive cofactor histone H1C [23,25]. MSX1 belongs to the NKL subclass of homeobox genes, many members of which are frequently and aberrantly activated in T-cell acute lymphoid leukemia as well as in lymphoid B-cell malignancies. In T-cell leukemia overexpression of MSX1 requires suppression of the inhibitory BMP-pathway, while in mantle cell lymphoma aberrantly enhanced histone acetylation and the TFs FOXC1 and HLXB9 are involved [25,26]. Physiologically, expression of MSX1 is restricted to the earliest stages of lymphopoiesis, undergoing downregulation in the ensuing differentiation steps of both B- and T-lymphoid lineages [23,26–28]. Furthermore, MSX1 is involved in the embryonal development of the neural plate border region (NPBR) and its descendants, comprising neural crest (NC) cells and placodes [29–31]. In this context several upstream regulators of MSX1 have been described including BMP-signalling and histone acetylation [32,33].

The aim of this study was the evaluation of factors and pathways which activate MSX1 expression in HL, focussing on homeodomain TF OTX2 which activates MSX1 in the NPBR [34]. We demonstrate that this factor contributes to lymphomagenesis via MSX1 activation and characterize OTX homeodomain factors as oncogenes in HL which deregulate the lymphoid differentiation factors ZHX1 and ZHX2.

## Materials and Methods

### Cell lines and treatments

HL cell lines are held by the DSMZ (Braunschweig, Germany). Cells were cultivated as described previously [35]. Treatments of cell lines were performed with 10 μg/ml Trichostatin A (TSA) (Sigma, Taufkirchen, Germany), with 10 μM IWR1 (R&D Systems, Wiesbaden, Germany), and with recombinant human Fibroblast Growth Factor 2 (FGF2) protein (R&D Systems) for 20 h. For the neutralization of FGF2 protein function in the culture medium we used anti-FGF2 antibody and an isotype control (R&D Systems). Gene specific siRNA oligonucleotides and AllStars negative Control siRNA (siControl) were obtained from Qiagen (Hilden, Germany). The expression construct for OTX2 was cloned into the vector pCMV6-XL5 and obtained from Origene (Wiesbaden, Germany). SiRNAs (80 pmol), expression constructs (2 μg), reporter constructs (1 μg), and luciferase reporter construct (200 ng) were transfected into 1x10^6^ cells by electroporation using the EPI-2500 impulse generator (Fischer, Heidelberg, Germany) at 350 V for 10 ms. Treated cells were subsequently harvested after 20 h.

### Polymerase chain-reaction (PCR) analyses

Total RNA was extracted using TRIzol reagent (Invitrogen, Darmstadt, Germany). Primary human material used in this study was commercially obtained—total human RNA isolated from peripheral blood mononuclear cells (PBC), thymus, lymph node (LN), and bone marrow (BM) from Clontech (Saint-Germain-en-Laye, France), and RNA from peripheral CD19-positive B-cells and CD3-positive T-cells from Miltenyi Biotec (Bergisch Gladbach, Germany). cDNA was synthesized from 5 μg RNA by random priming using Superscript II (Invitrogen).

Real-time quantitative gene expression analysis (RQ-PCR) was performed with the 7500 Real-time System, using commercial buffer and primer sets (Applied Biosystems, Darmstadt, Germany). Quantification of MSX1 was performed as described recently [23]. For normalization of expression levels we analyzed the transcript of TATA box binding protein (TBP). Quantitative analyses were performed in triplicate. The standard deviations are presented in the figures as error bars. The statistical significance was assessed by Student´s T-Test. The calculated p-values are indicated by asterisks (* p<0.05, ** p<0.01, *** p<0.001, n.s. no significance).

### Protein analyses

Western blots were generated by the semi-dry method. Proteins obtained from cell line lysates using SIGMAFast protease inhibitor cocktail (Sigma) were transferred onto nitrocellulose membranes (Bio-Rad, München, Germany) and blocked with 5% dry milk powder dissolved in phosphate-buffered-saline buffer (PBS). The following antibodies were used: alpha-Tubulin (Sigma), OTX1 (Santa Cruz Biotechnology, Heidelberg, Germany), OTX2 (Abcam, Cambridge, UK), and ZHX1 (Santa Cruz Biotechnology). For loading control the blots were stained with Poinceau (Sigma) and then detection of alpha-Tubulin (TUBA) was performed. Secondary antibodies were linked to peroxidase for detection by Western-Lightning-ECL (Perkin Elmer, Waltham, MA, USA). Documentation was performed using the digital system ChemoStar Imager (INTAS, Göttingen, Germany).

Quantification of FGF2 protein in cell culture supernatants was performed as follows: log-phase cells were washed twice in PBS and cultivated in fresh medium at 1x10^6^ cells per ml and supernatants harvested after 24 h. ELISA was performed using the Human FGF basic Quantikine ELISA Kit (R&D Systems) and an ELISA reader (Thermo Electron, Vantaa, Finland).

### Chromosomal and genomic analyses

Chromosomal analysis by fluorescent in-situ hybridization (FISH) was performed as described previously [36]. RP11-BAC clones were obtained from BacPac Resources, Children´s Hospital Oakland Research Institute (CA, USA), insert DNA harvested using the Big BAC DNA Kit (Princeton Separations, Adelphia, NJ, USA) and directly labelled by nick translation with dUTP-fluors (Dyomics, Jena, Germany). Fluorescent images were captured and analyzed with an Axio-Imager microscope (Zeiss, Göttingen, Germany) configured to a dual Spectral Imaging FISH system (Applied Spectral Imaging, Neckarhausen, Germany).

For genomic profiling of the cell lines KM-H2 and U-HO1 genomic DNA was prepared by the Qiagen Gentra Puregene Kit (Qiagen). Labelling, hybridization and scanning were performed at the Genome Analytics Facility, Helmholtz Centre for Infection Research (Braunschweig, Germany), according to the manufacturer´s protocols (Affymetrix, High Wycombe, UK). Data were interpreted using the Chromosome Analysis Suite software version 2.0.1.2 (Affymetrix). Genomic profiling of HDLM-2 and L-1236 was performed using datasets from Gene Expression Omnibus (GEO), GSM381298 for HDLM-2 and GSM381297 for L-1236, the Affymetrix Genotyping Console GTC Software version 4.0 (Affymetrix), and visualized by the Affymetrix GTC-Browser program as described recently [22].

### Expression profiling

Gene expression microarray profiling data were obtained using the HG U133 Plus 2.0 gene chip (Affymetrix). Datasets for HL cell lines HDLM-2, KM-H2, L428, L-540, L-1236 and SUP-HD1 were generated by Prof. Andreas Rosenwald (Institute of Pathology, University of Würzburg, Germany) as described previously [37]. Datasets for untreated HL cell line U-HO1 and for KM-H2 cells treated with siRNA were generated by Dr. Robert Geffers (Genome Analytics Facility, Helmholtz Centre for Infection Research, Braunschweig, Germany). The datasets for these 7 untreated HL cell lines were transformed into log2-values and combined in one table (**S1 Table**). To parse biological function of shortlisted genes, gene set enrichment analysis was performed using DAVID bioinformatics resources [38].

For in silico expression analysis of primary samples from HL patients and normal B-cell subtypes we used the GEO dataset GSE12453 supplemented with online analysis tools at the National Center for Biotechnology Information [39]. The primary material for this dataset was obtained from microdissected tumor cells, comprising 12 classical HL and 5 nodular lymphocyte-predominant HL cases. For in silico expression analysis of BM in comparison to resting B-cell samples we used dataset GSE7307 which comprises profiling data of most human tissues. Additional analyses of expression data were performed using Microsoft Excel and R-based statistical tools (http://cran.r-project.org/).

### Reporter gene assay

For creation of the reporter gene construct we combined a reporter with a regulatory genomic fragment derived from the upstream region of ZHX1 located between -2108 bp and -942 bp, containing three potential binding sites for OTX2 [40,41]. We cloned genomic PCR products of the corresponding ZHX1 upstream region (regulator) and of the HOXA9 gene, comprising exon1-intron1-exon2 (reporter), into the *Hind*III/*Bam*HI and *Eco*RI sites, respectively, of the expression vector pcDNA3 downstream of the CMV enhancer [23]. The oligonucleotides used for the amplification of the ZHX1-regulator were obtained from MWG Eurofins (Ebersberg, Germany) and the sequences were as follows: forward 5´-CGAAGCTTGAGGTTAGGAGTTCGCAACC-3´, reverse 5´-CCGGATCCCAGAGCTCCGCTGAGCCACAG-3´. The construct was validated by sequence analysis (MWG Eurofins). Commercial HOXA9 and TBP assays were used for RQ-PCR to quantify the spliced reporter-transcript, corresponding to the regulator activity. A cotransfected commercial luciferase construct served as transfection control and was quantified by the Luciferase Assay System (Promega, Mannheim, Germany) using the luminometer Lumat LB9501 (Berthold Technologies, Bad Wildbad, Germany).

## Results

### Expression of OTX homeobox genes in HL

**Fig 1:**
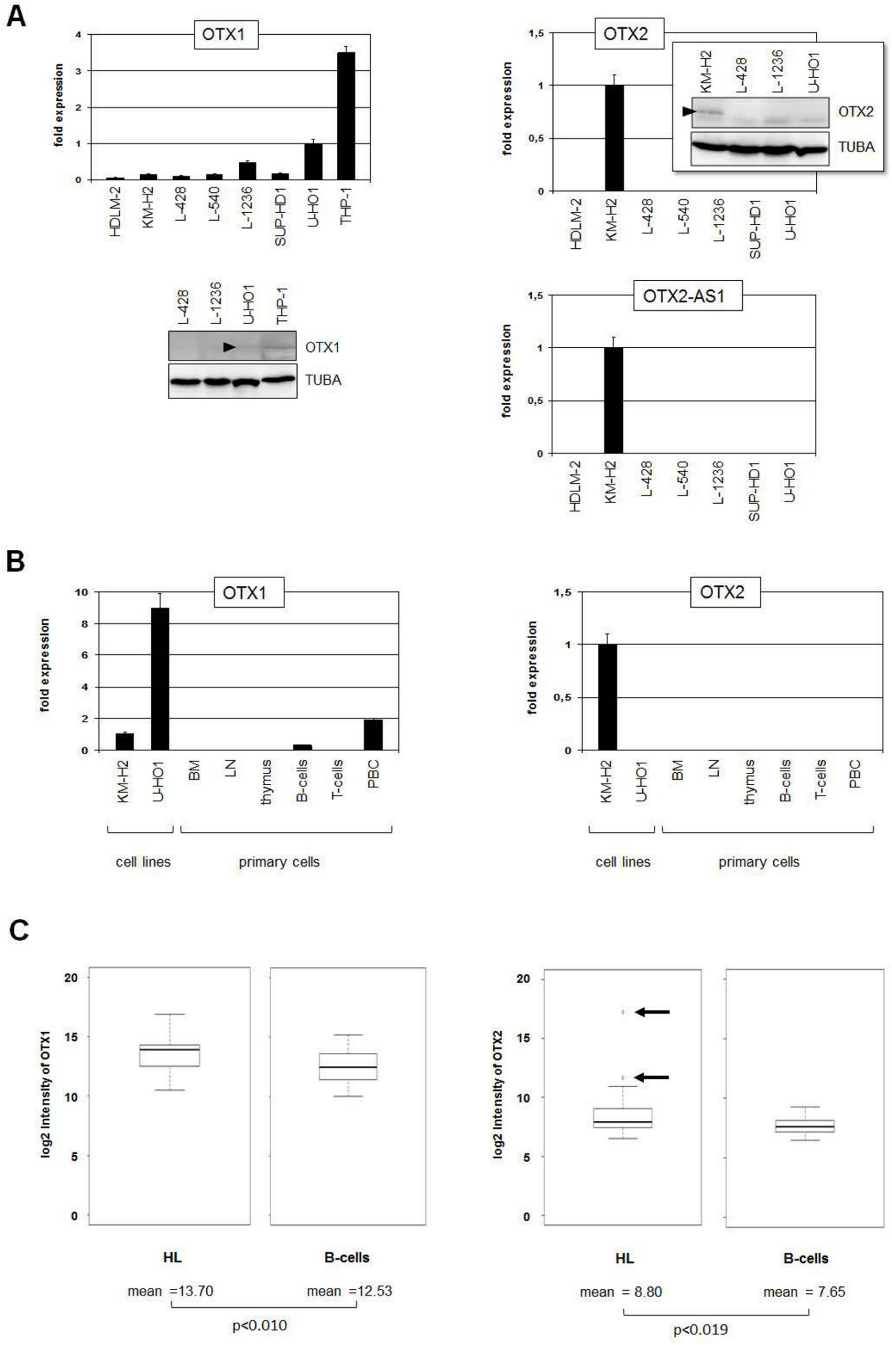
OTX expression in HL cell lines and patients. (A) RQ-PCR analysis of OTX1 (left), OTX2 (middle) and OTX2-AS1 (right) in HL cell lines. The highest OTX1 expression level is visible in U-HO1. KM-H2 exclusively expresses OTX2 and OTX2-AS1. Western blot analysis of OTX2 demonstrates OTX2 protein in KM-H2 (insert in the middle), confirming the RQ-PCR data. Of note, the faint bands visible in all probes represent unspecific background signals produced by this antibody. (B) RQ-PCR analysis of primary hematopoietic samples in comparison to HL cell lines of OTX1 (left) and OTX2 (right). While OTX1 shows low expression levels in PBC and B-cells, OTX2 is undetectable in primary cells, indicating ectopic expression in KM-H2. (C) In silico expression analysis of OTX1 (left) and OTX2 (right) in HL patient samples in comparison to normal B-cells shows significantly higher expression levels in HL cells (GSE12453). In addition, 12% (2/17) of HL patient samples showed OTX2 overexpression (arrows).

We started our study by transcript quantification of OTX family members OTX1 and OTX2 in seven HL cell lines. OTX1 transcripts were detected in all HL cell lines analyzed with U-HO1 showing the highest levels (**Fig 1A**). However, THP-1 cells were used as positive control expressing 3-times higher levels as U-HO1. Western blot analysis detected just a faint signal in U-HO1, demonstrating low OTX1 expression in HL cell lines (**Fig 1A**). Expression of OTX2 was exclusively detected in one HL cell line, namely KM-H2, at both the RNA and protein levels, indicating aberrant activity (**Fig 1A**). The antisense gene OTX2-AS1 is located directly upstream of OTX2 and was also exclusively expressed in KM-H2 (**Fig 1A**). To examine the expression of OTX1 and OTX2 in normal hematopoietic cells and tissues we performed RQ-PCR analyses in BM, LN, thymus, B-cells, T-cells and PBC obtained from healthy donors. While OTX1 expression was only weakly expressed in PBC and B-cells, OTX2 transcripts were not detected in any primary hematopoietic sample (**Fig 1B**). To analyze OTX gene expression in primary HL patient samples we used the GEO data set GSE12453 [39]. Accordingly, HL patients expressed significantly higher levels of OTX1 and OTX2 as compared to normal B-cells. However, differences in OTX1 expression levels were minor. But OTX2 demonstrated conspicuously enhanced expression levels in 12% (2/17) of HL patients (**Fig 1C**, **S1 Fig**). The expression levels of OTX2-AS1 in HL patients and B-cells overlapped, suggesting absence of any functional relation to OTX2 gene activity (**S1 Fig**). Taken together, we identified aberrantly enhanced expression of homeobox genes OTX1 and OTX2 in subsets of both HL cell lines and HL patients.

### Chromosomal analysis of OTX gene loci in HL cell lines

**Fig 2:**
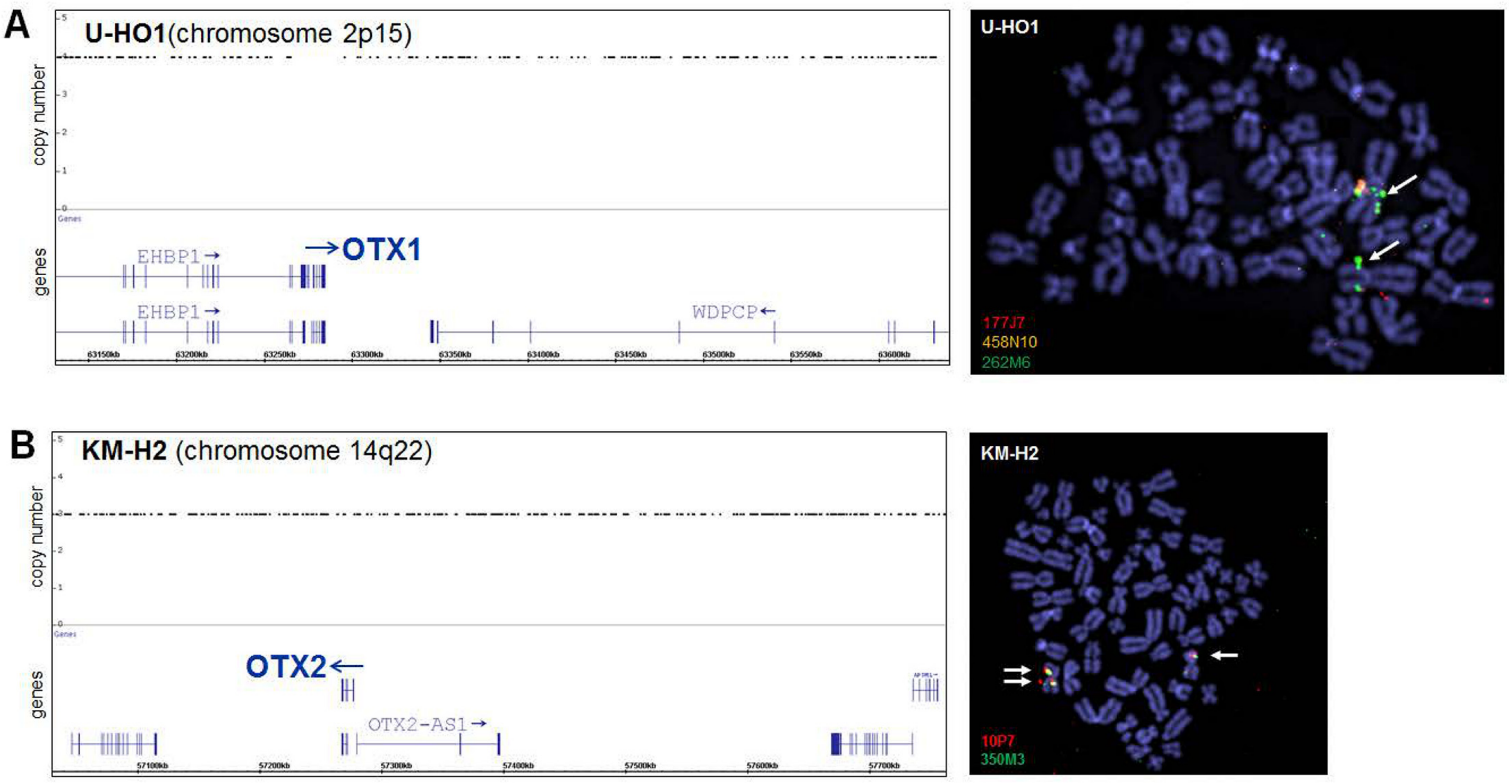
Genomic and chromosomal analysis of OTX loci. HL cell lines U-HO1 (A) and KM-H2 (B) were analyzed by genomic profiling and FISH. Loci for OTX1 (2p15) in U-HO1 and for OTX2 (14q22) in KM-H2 are duplicated as shown by copy number (left) and FISH analyses (right), demonstrating duplicated and enhanced signals, respectively (arrows). BAC-probes cover (458N10) and flank (177J7, 262M6, 10P7, 350M3) the analyzed genes and are indicated in their labelling fluor colors.

To check if chromosomal aberrations in HL cell lines U-HO1 and KM-H2 may contribute to the deregulated expression of the OTX genes observed therein we performed genomic profiling and FISH analyses. We detected copy number gains of the loci for OTX1 located at chromosomal band 2p15 (4-fold) and OTX2 at 14q22 (3-fold) in the respective HL cell lines by genomic profiling (**Fig 2**). FISH analysis confirmed amplification of OTX1 in U-HO1 and duplication of OTX2 in KM-H2 but excluded translocation breakpoints nearby (**Fig 2**). These data indicate that genomic rearrangements resulted in elevated copy numbers of both OTX loci, correlating with their enhanced expression levels.

### Expression profiling analyses

While OTX1 expression has been described in small subsets of normal germinal center B-cells in addition to high levels in subsets of aggressive non-HL, OTX2 was detected neither in normal B-cells nor in B-cell lymphomas [42,43]. Although we have identified ectopic expression of OTX2 just in subsets of HL this homeobox gene may deregulate general oncogenic targets or pathways and thus contribute to lymphomagenesis. We focused our study on OTX2 which may deepen our understanding and reveal undisclosed oncogenic function(s) in this heterogeneous type of lymphoma.

To identify potential OTX2 activators we performed comparative expression profiling of HL cell lines, KM-H2 (OTX2-positive) in relation to HDLM-2, L-428, L-540, L-1236, and SUP-HD1 (all OTX2-negative). This comparison generated lists of the top 1000 over/under-expressed genes in KM-H2 (**S2 and S3 Tables**). Gene set enrichment analysis of the top 1000 overexpressed genes indicted several cellular processes, notably cytokine-signalling, MAPK-activity and FGF-pathway (**S2 Fig**). Conspicuously expressed genes included RTN1 (histone deacetylase inhibitor), and FGF2, FGFR2 and WNT5A (signalling pathways). Thus, chromatin alteration via histone acetylation and FGF- and WNT-signalling pathways may contribute to aberrant OTX2 expression in HL cell line KM-H2.

To identify potential OTX2 target genes we performed gene expression profiling of KM-H2 cells treated for siRNA-mediated knockdown of OTX2 (**S3 Table**). The data confirmed the reduction of OTX2 expression and revealed elevated expression of ZHX1 (TF) and reduction of CTNNBIP1 (WNT-signalling inhibitor). These candidate genes were analyzed in more detail as described below.

### Analyses of potential upstream regulators of OTX2

**Fig 3:**
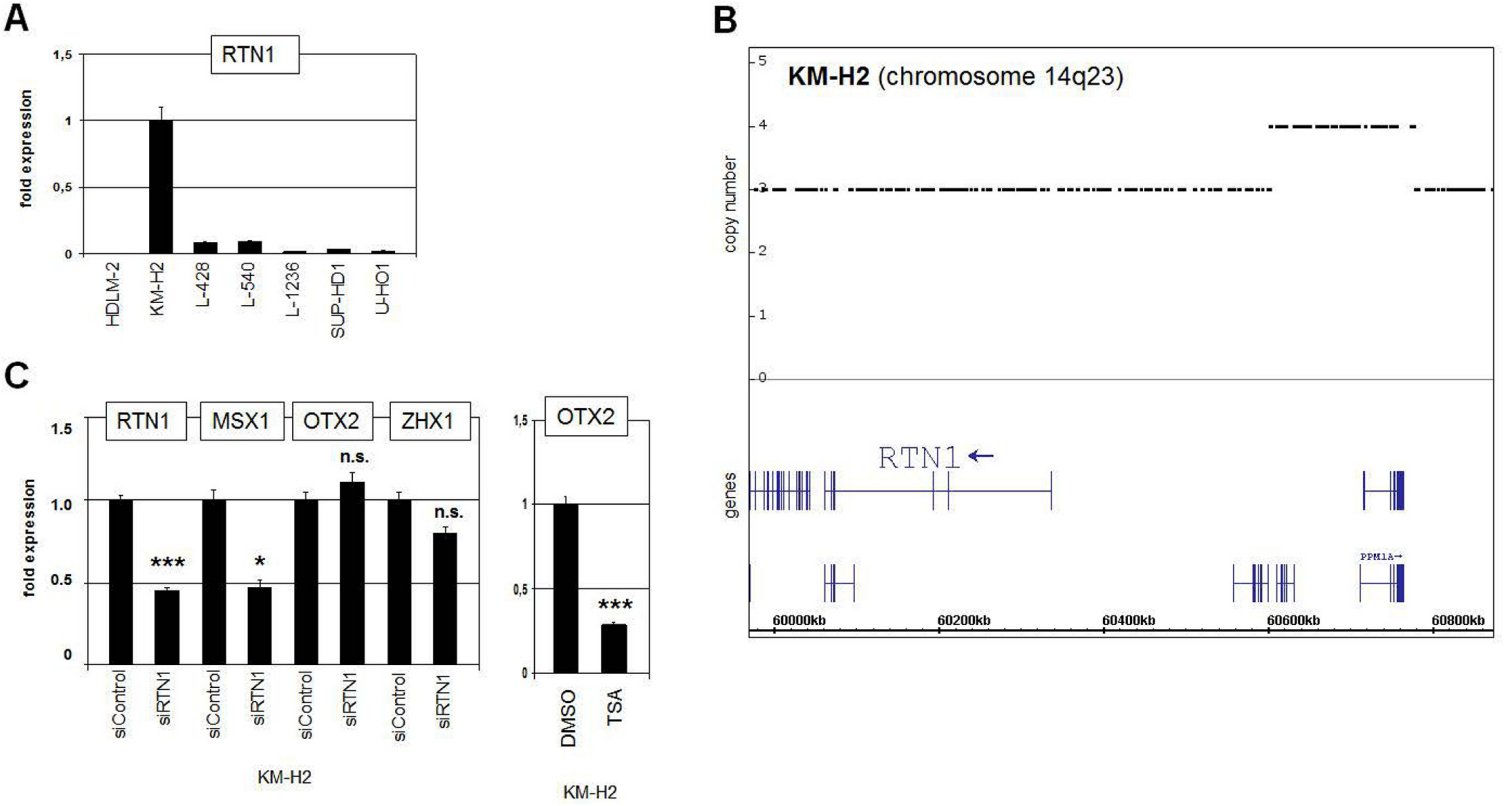
Analysis of HDAC inhibitor RTN1. (A) RQ-PCR analysis of RTN1 expression in HL cell lines shows enhanced transcription in KM-H2, confirming the expression profiling data. (B) Genomic profiling data of chromosome 14 for KM-H2 indicate copy number gain of RTN1 and partial duplication of the upstream regulatory region of RTN1 at 14q23. (C) RQ-PCR analysis of KM-H2 cells treated for siRNA-mediated knockdown of RTN1 (left). Reduced expression of RTN1 was confirmed, while the expression of OTX2 did not change. Reduced expression of MSX1 indicated regulation by histone acetylation and HDAC inhibitor RTN1. Treatment of KM-H2 with HDAC inhibitor TSA resulted in reduced expression of OTX2 (right), indicating indirect regulation by acetylation of non-histone proteins.

Chromatin alteration via histone acetylation activates several genes encoding developmental regulators including MSX1 and OTX2 [25,33,44]. Quantification of RTN1 transcripts revealed enhanced expression in KM-H2 as compared to HL control cell lines (**Fig 3A**), confirming the initial comparative profiling results. Genomic profiling data indicated copy number gain of RTN1 (3-fold) and partial duplication of the far upstream region of RTN1 at 14q23 (**Fig 3B**), which may have contributed to its elevated expression in KM-H2. RTN1 encodes an inhibitor of histone deacetylases (HDACs), suggesting a regulatory impact via enhanced histone acetylation [45]. However, siRNA-mediated knockdown of RTN1 in KM-H2 cells resulted in reduced expression of MSX1 but not of OTX2, discounting a regulative impact on this gene (**Fig 3C**). Treatment of KM-H2 with HDAC inhibitor TSA resulted in reduced expression levels of OTX2, indicating indirect regulation via acetylation of non-histone factors (**Fig 3C**). This effect has been also observed in developing tissues of the eye and the brain [46,47], suggesting corresponding regulatory mechanisms.

**Fig 4:**
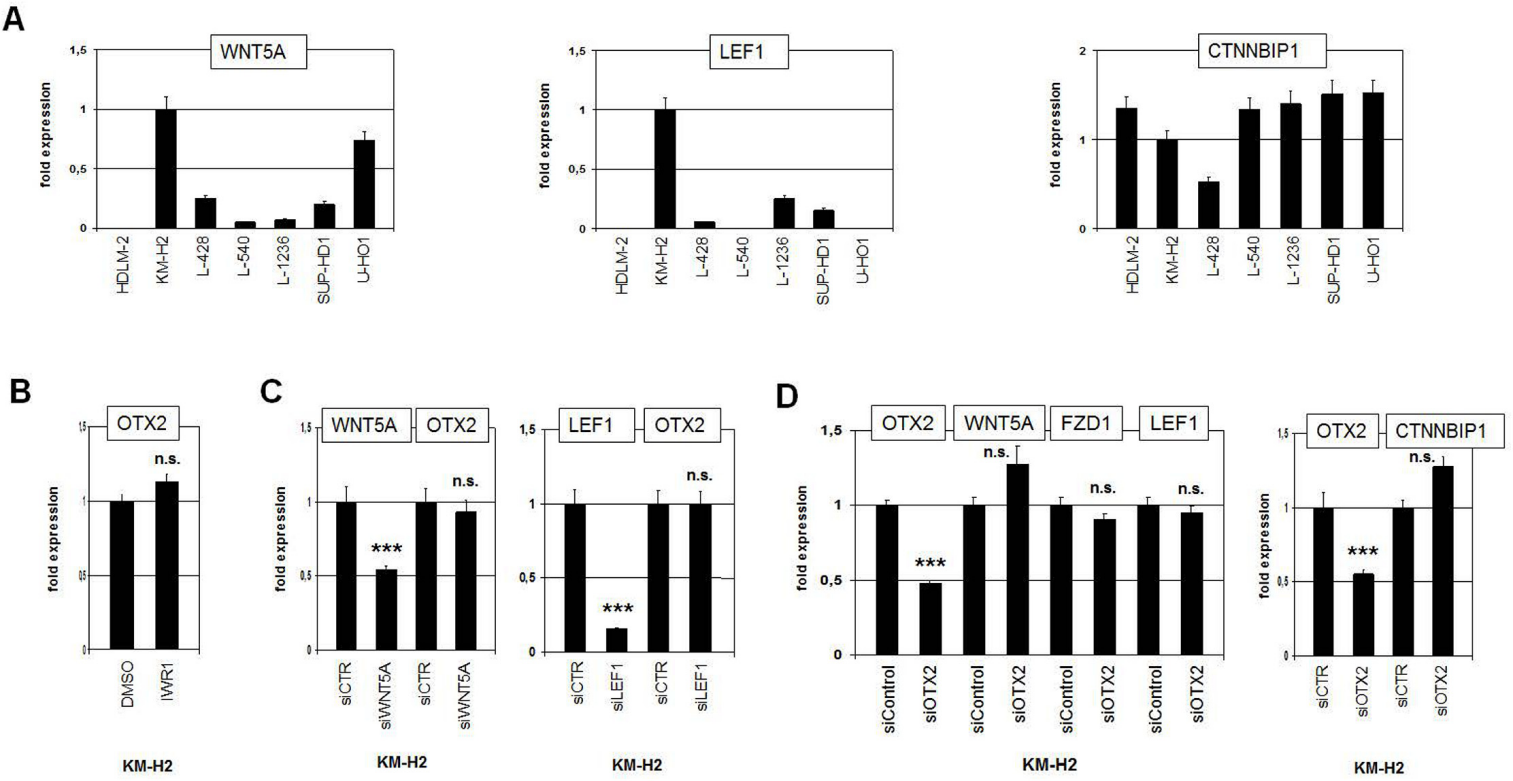
WNT-signalling pathway in HL. (A) RQ-PCR analyses of WNT5A (left), LEF1 (middle), and CTNNBIP1 (right) in HL cell lines indicate enhanced activity of the WNT-pathway in KM-H2 cells. (B) RQ-PCR analysis of OTX2 in KM-H2 (left) and of OTX1 in U-HO1 (right) after treatment with WNT-signalling inhibitor IWR1 shows no change in expression levels. (C) RQ-PCR analysis of OTX2 in KM-H2 cells after siRNA-mediated knockdown of WNT5A (left) and of LEF1 (right). While the expression levels of WNT5A and LEF1 were significantly reduced, transcript levels of OTX2 remained unchanged, indicating absence of regulatory input. (D) RQ-PCR analysis of WNT5A, FZD1, LEF1 (left), and of CTNNBIP1 (right) after siRNA-mediated knockdown of OTX2. The resultant alterations in transcript levels are not significant, indicating absence of regulatory input of OTX2 on these WNT-pathway components.

HL is associated with deregulation of multiple signalling pathways. However, few investigations of the WNT-pathway in this disease have been reported so far [48]. RQ-PCR analysis of candidate WNT-pathway components in HL cell lines demonstrated enhanced expression levels of WNT5A (ligand) and LEF1 (downstream TF) and reduced levels of CTNNBIP1 (negative regulator) in KM-H2 (**Fig 4A**), suggesting aberrant signalling activity in this cell line. In silico HL patient sample data show enhanced expression of WNT5A, LEF1, FZD7 and reduced levels of CTNNBIP1 (**S3 Fig**), supporting the cell line data. However, treatment of KM-H2 with WNT-pathway inhibitor IWR1, siWNT5A or siLEF1 showed no alterations in OTX2 expression (**Fig 4B and 4C**), discounting WNT-signalling regulation in HL. In addition, siRNA-mediated knockdown of OTX2 in KM-H2 showed no significant effect on the expression levels of WNT5A, FZD1, LEF1 or CTNNBIP1, demonstrating that OTX2 does not activate these WNT-pathway components (**Fig 4D**). Thus, no mutual regulation of OTX2 and WNT-signalling was detected, although both OTX2 and this pathway evidence aberrant activation in HL subsets.

**Fig 5:**
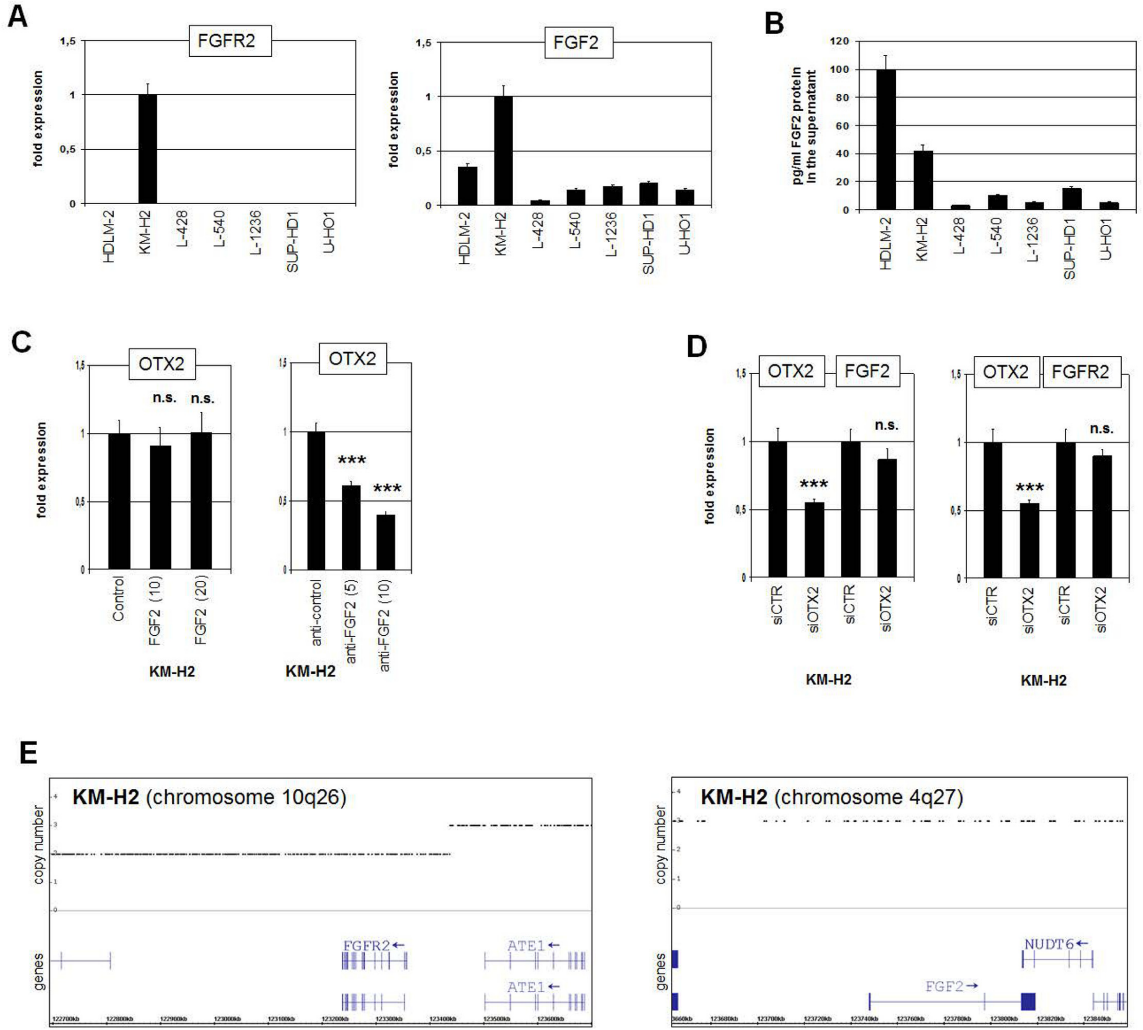
FGF-signalling pathway in KM-H2. (A) RQ-PCR analyses of FGFR2 (left) and FGF2 (right) in HL cell lines indicate constitutive activity of the FGF2-pathway in KM-H2 cells. (B) FGF2 protein quantification in the supernatants of the HL cell lines by ELISA detected elevated levels in HDLM-2 and KM-H2. (C) RQ-PCR analysis of OTX2 expression in KM-H2 cells after treatment with 10 or 20 ng/ml FGF2 protein (left) and 5 or 10 ng/ml anti-FGF2 antibody (right). While increasing FGF2 protein in the culture medium showed no effect, suppression of FGF2-activity via inhibitory antibody reduced OTX2 expression, supporting the activatory role for FGF2-signalling in OTX2 expression. (D) RQ-PCR analysis of FGF2 (left) and of FGFR2 (right) after siRNA-mediated knockdown of OTX2 in KM-H2 cells. The data discounted stimulation of these genes by OTX2. (E) Genomic profiling data for KM-H2 of chromosome 10 (left) indicate partial duplication of the upstream regulatory region of FGFR2 at 10q26, and of chromosome 4 (right) gain of the FGF2 locus at 4q27.

To gauge the impact of FGF2-signalling we first analyzed the expression of FGFR2 (receptor) and FGF2 (ligand) in HL cell lines. RQ-PCR results confirmed high transcript levels of FGFR2 and FGF2 in KM-H2 (**Fig 5A**). Moreover, ELISA data demonstrated high FGF2 protein amounts in the supernatants of the cell lines HDLM-2 and KM-H2 (**Fig 5B**), indicating active autocrine FGF2-signalling in KM-H2. Enhanced expression of FGF2, FGFR2 and FGFR3 was detected in HL patient samples in silico (**S3 Fig**), supporting a general activation of this pathway in HL as described recently [49,50]. Treatment of KM-H2 cells with recombinant FGF2 protein yielded no change in OTX2 transcription (**Fig 5C**), consistent with saturated autocrine FGF2 activity in this cell line. In contrast, treatment of KM-H2 with inhibitory FGF2 antibody resulted in concentration dependent reduction of OTX2 expression (**Fig 5C**), indicating stimulation by this pathway. However, siRNA-mediated knockdown of OTX2 left FGF2 and FGFR2 expression levels unaltered in KM-H2 (**Fig 5D**), discounting a role for this factor in regulating these pathway components. But genomic profiling data demonstrated copy number changes at the loci of FGFR2 and FGF2 (**Fig 5E**), indicating partial duplication of the upstream regulatory region of FGFR2 at 10q26 and a gain of the locus of FGF2 at 4q27. These genomic alterations may contribute to elevated expression of FGFR2 and FGF2 in KM-H2. Thus, aberrantly activated FGF2-signalling pathway mediates OTX2 expression in HL cells.

### Identification of transcription factors regulated by OTX2

**Fig 6:**
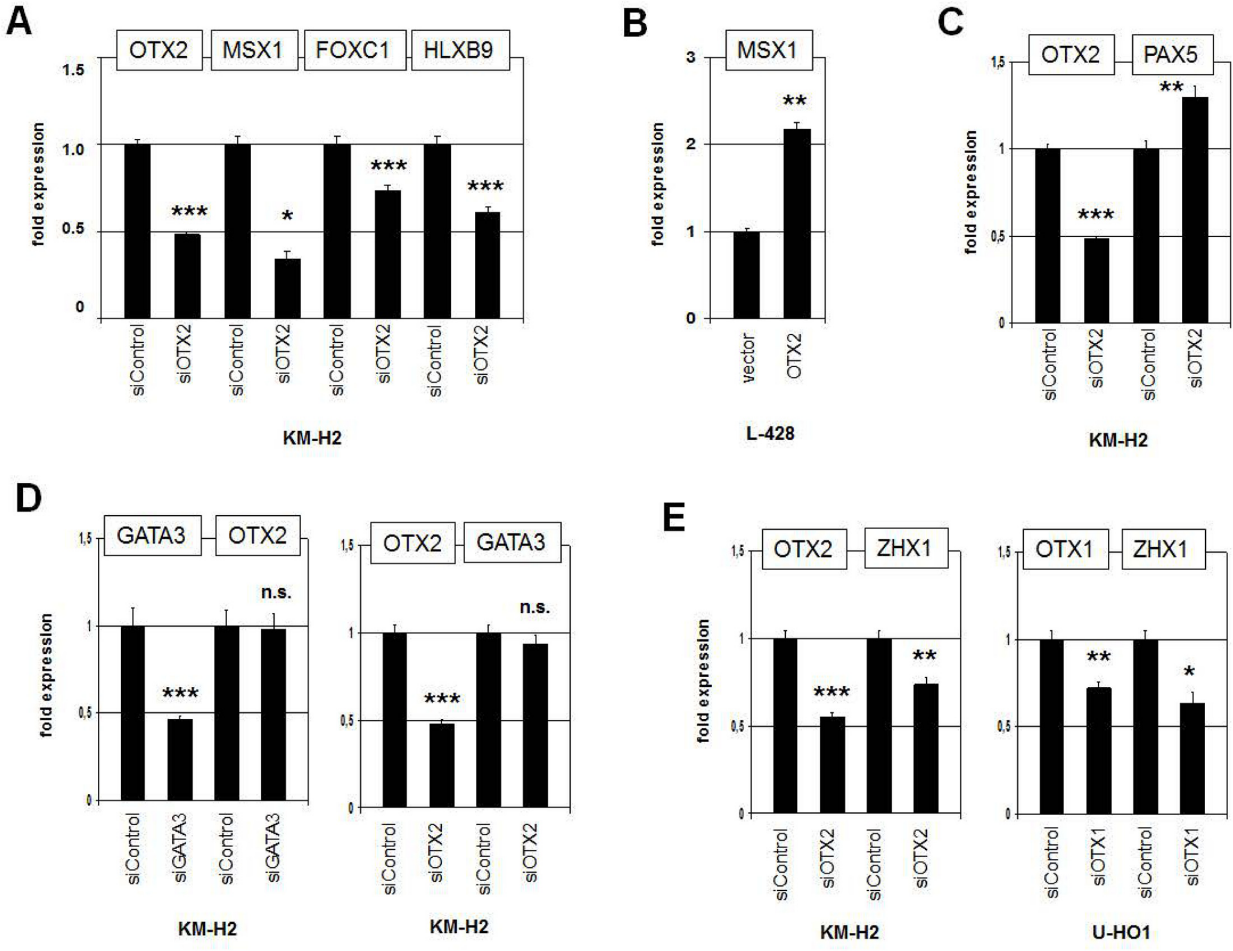
Analysis of selected TFs regulated by OTX2. (A) RQ-PCR analysis of MSX1, FOXC1 and HLXB9 after siRNA-mediated knockdown of OTX2 in KM-H2 cells. The data indicate that OTX2 activates the transcription of these TF genes. (B) RQ-PCR analysis of MSX1 expression after forced expression of OTX2 in L-428 cells, confirming an activating input of OTX2. (C) RQ-PCR analysis of PAX5 expression after siRNA-mediated knockdown of OTX2 in KM-H2 cells, showing slightly increased expression levels. (D) RQ-PCR analysis of GATA3 and OTX2 after their siRNA-mediated knockdown, indicating absence of mutual regulation. (E) RQ-PCR analysis of ZHX1 after siRNA-mediated knockdown of OTX2 in KM-H2 (left) and of OTX1 in U-HO1 cells (right). The data demonstrate that both OTX1 and OTX2 activate expression of ZHX1.

The initial aim of our study was the identification of potential activators of homeobox gene MSX1 which is deregulated in HL [23]. To check if OTX2 regulates MSX1 in HL cell line KM-H2 we performed siRNA-mediated knockdown of OTX2. Subsequent quantification of MSX1 expression demonstrated decreased transcript levels (**Fig 6A**), consistent with activation of MSX1 by OTX2 in HL. Moreover, forced expression of OTX2 in OTX2-negative L-428 cells enhanced MSX1 expression about twofold (**Fig 6B**). Additional TFs aberrantly overexpressed in HL cell lines include FOXC1 and HLXB9 [26,51]. Both genes showed reduced expression levels after OTX2 knockdown (**Fig 6A**), supporting their transcriptional activation by OTX2 in KM-H2. Consistently, we identified in both genes potential binding sites for OTX2 in their regulatory regions. FOXC1 contains 6 sites in the far upstream region between -46.2 and -27.8 kb. HLXB9 contains 2 sites in the promoter region at -3.5 kb. In contrast, after OTX2 knockdown the expression of the B-cell factor PAX5 rose perceptibly (**Fig 6C**). This effect is probably indirect because PAX5 is suppressed by FOXC1 in HL [26], which is in turn activated by OTX2. Finally, GATA3 has been shown both to be aberrantly expressed in HL and to regulate OTX2 during embryogenesis in a conserved network [14,52,53]. However, siRNA-mediated knockdown experiments excluded mutual regulation of GATA3 and OTX2 in this malignancy (**Fig 6D**).

Our profiling data revealed reduced expression of ZHX1 in KM-H2 after siRNA-mediated knockdown of OTX2 (**S4 Table**), suggesting that ZHX1 represents a potential target gene. RQ-PCR analysis confirmed decreased expression levels in these treated cells (**Fig 6E**), showing that OTX2 activates ZHX1. The same result was obtained after knockdown of OTX1 in U-HO1 (**Fig 6E**), demonstrating that ZHX1 represents a target gene of both OTX factors in HL. Raised ZHX1 and reduced ZHX2 levels, as described recently [22], were also observed in silico in primary HL patient samples (**S3 Fig**), consolidating the cell line data and the clinical relevance of these genes. While ZHX2 is a B-cell specific Zn-finger TF encoding gene [22,24], the closely related TF ZHX1 has been described in developing brain and in murine cell lines representing bone marrow stroma and T-cells [54,55]. Therefore, we analyzed ZHX1 in hematopoietic cells in more detail.

**Fig 7:**
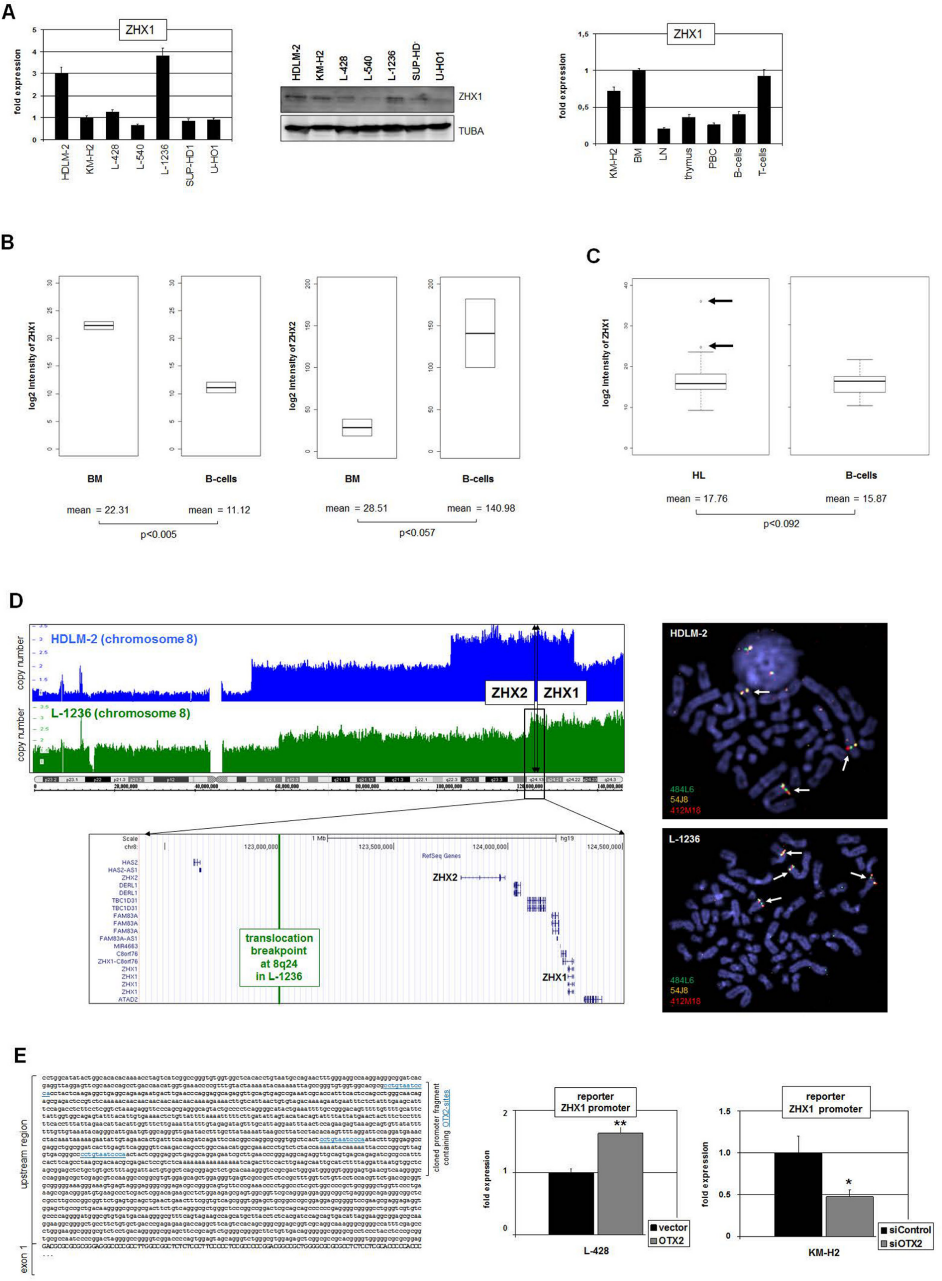
Deregulated expression of ZHX1 in HL. (A) RQ-PCR (left) and Western blot (middle) analyses of ZHX1 in HL cell lines consistently indicate constitutive activity and enhanced expression in HDLM-2 and L-1236 cells. RQ-PCR analysis of ZHX1 in primary hematopoietic samples (right) shows highest levels in BM and T-cells and reduced expression in LN, thymus, PBC and B-cells. The ZHX1 expression in KM-H2 is elevated and corresponds to the levels observed in BM and T-cells. (B) In silico expression analysis of ZHX1 (left) and ZHX2 (right) in primary BM and B-cell samples (GSE7307). The data show significantly different expression levels for both genes. The expression level of ZHX1 is reduced while that of ZHX2 is elevated in B-cells as compared to BM. (C) In silico expression analysis of ZHX1 in primary samples obtained from HL patients and from B-cells of healthy donors (GSE12453). The expression level of ZHX1 is significantly increased in HL samples as compared to B-cells and shows overexpression in 12% (2/17) HL patients (arrows). (D) Copy number analyses of chromosome 8 (left) and FISH analyses (right) in HL cell lines HDLM-2 and L-1236. The gene loci of ZHX1 and ZHX2 are indicated, showing their localization in amplified regions of both cell lines. While both genes are entirely amplified in HDLM-2, L-1236 contains a chromosomal breakpoint in the upstream region of ZHX2. The FISH results confirm the copy number data, indicating the signals at the gene locus of ZHX1 by arrows. The BACs used were 484L6 (ZHX2), 54J8 (ZHX1) and 412M18 (flanking). (E) Reporter gene assay analyzing the promoter region of ZHX1, containing three potential binding sites for OTX2 as indicated in blue (left). RQ-PCR analysis of the reporter gene in L-428 cells after forced expression of OTX2 (right, above) and in KM-H2 cells after siRNA-mediated knockdown of OTX2 (right, below). Collectively, the data show that OTX2 activates directly the expression of ZHX1 in HL cells.

RQ-PCR and Western blot analysis of ZHX1 consistently demonstrated varying expression levels in all HL cell lines with HDLM-2 and L-1236 showing peak values (**Fig 7A**). In primary hematopoietic samples highest transcript levels were present in BM and T-cells, while LN, PBC and B-cells expressed reduced levels (**Fig 7A**). These data indicate that while ZHX1 plays a role in early hematopoiesis and remains active in T-cells, it is downregulated in B-cells. In silico analysis of primary samples obtained from BM and from resting B-cells (GSE7307) supported these observations, confirming that ZHX1 is downregulated in B-cells accompanied by upregulation of ZHX2 (**Fig 7B**). Thus, ZHX2 plays a role in B-cell development and ZHX1 in T-cell differentiation. In silico expression analysis of ZHX1 demonstrated higher levels in HL patients than in B-cells obtained from healthy donors (GSE12453). Of note, this difference is not significant (p<0.092). However, 12% (2/17) of the patients showed aberrantly enhanced expression levels of ZHX1 (**Fig 7C**), supporting the cell lines data and an oncogenic potential in the clinical setting.

Interestingly, the genes ZHX1 and ZHX2 are located closely together at chromosomal band 8q24, a sign of evolutionary gene duplication. Genomic profiling data and FISH analysis of ZHX1 in HDLM-2 and L-1236 indicated oncogenomic duplication of this gene in both cell lines (**Fig 7D**), which may contribute to overexpression in these cells. While L-1236 contains a chromosomal break in the upstream region of ZHX2 [22], in HDLM-2 the duplication comprises both genes in toto. However, both cell lines show reduced expression levels of ZHX2. In L-1236 the transcription is disturbed due to chromosomal aberration t(4;8)(q27;q24), in HDLM-2 is no protein detectable despite enhanced transcription, indicating post-transcriptional repression [22].

Finally, sequence analysis of the ZHX1 gene revealed three potential OTX binding sites in the upstream promoter region covering bicoid consensus sites [40,41]. Therefore, we designed a reporter gene assay analyzing the impact of OTX2 on this regulatory part of the ZHX1 gene. Overexpression of OTX2 in L-428 resulted in activation of the reporter gene, while siRNA-mediated reduction of OTX2 in KM-H2 yielded decreased reporter gene activity (**Fig 7E**), indicating that OTX2 binds to the promoter region of ZHX1 and activates the transcription directly.

## Discussion

**Fig 8:**
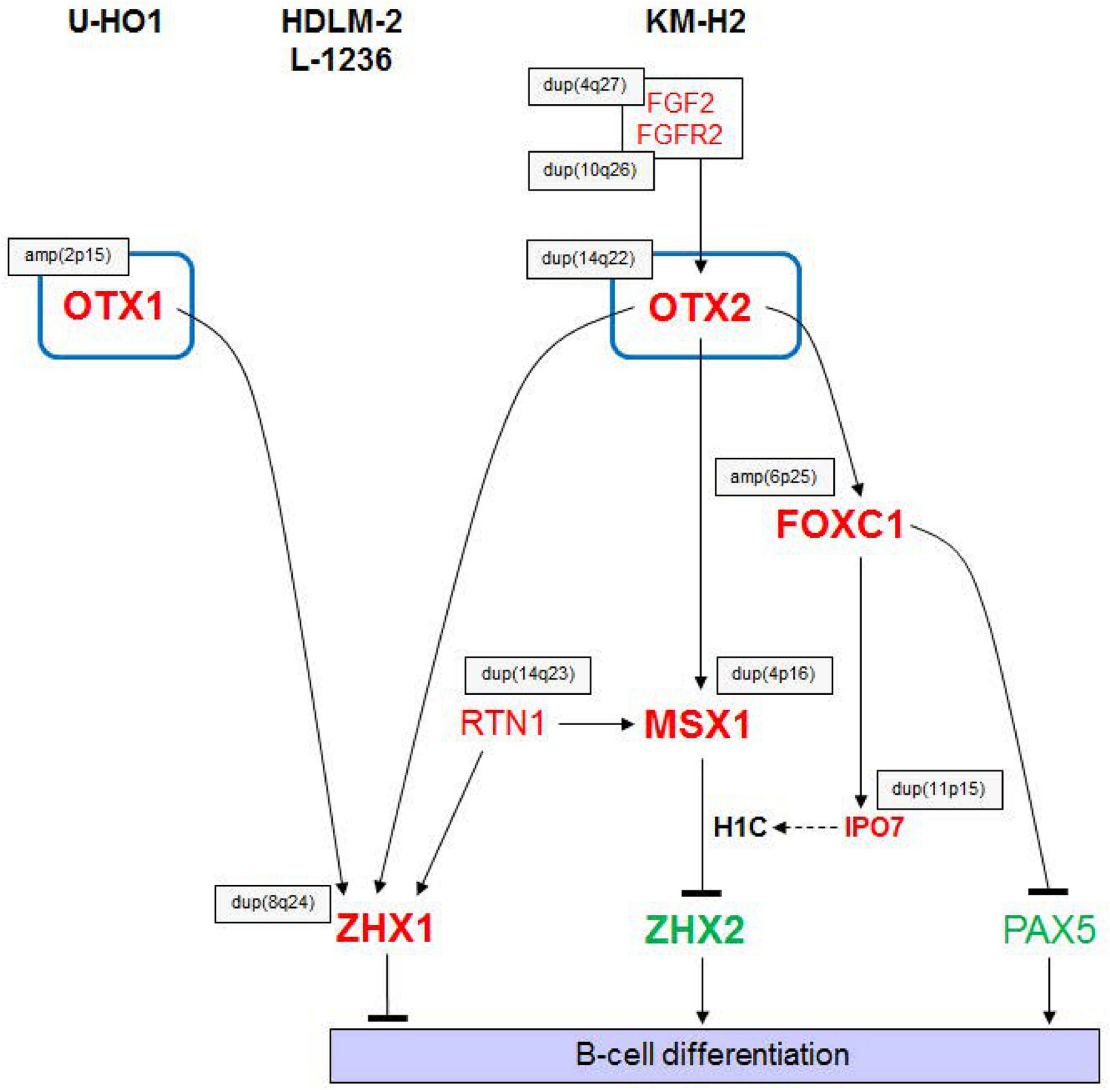
Aberrant gene regulatory network. The diagram summarizes the results obtained in this and previous studies in HL cell lines. OTX1 and OTX2 activate the expression of ZHX1. Additionally, OTX2 activates MSX1 and FOXC1 which subsequently inhibit ZHX2 and PAX5, respectively [23,26]. Most genes in this diagram are targeted by genomic/chromosomal aberrations (indicated by grey boxes). The main output of this aberrant regulatory network mediates suppression of B-cell differentiation. Overexpressed genes are illustrated in red, underexpressed genes in green.

Our results showing aberrant TF activities in HL are summarized in a gene regulatory network (GRN) (**Fig 8**). Homeobox genes OTX1 and OTX2 are targeted by copy number alterations in U-HO1 and KM-H2, respectively. Additionally, OTX2 expression is supported by the aberrantly activated FGF2-pathway in KM-H2. Genes encoding the TFs MSX1 and FOXC1 are located downstream of OTX2 which, as we recently showed, mediate repression of ZHX2 and PAX5, respectively [23,26]. Furthermore, the expression of ZHX1 is activated by OTX1 in U-HO1 and by OTX2 in KM-H2. Enhanced ZHX1 expression in the cell lines HDLM-2 and L-1236 correlates with chromosomal duplications therein, indicating multiple mechanisms of aberrant ZHX1 activation in HL. Reduced expression of the B-cell genes PAX5 and ZHX2 together with activation of the T-cell gene ZHX1 may contribute to the deregulated B-cell differentiation phenotype in this malignancy. The gene deregulations identified here were confirmed by data obtained from primary cells originating from HL patients, supporting their clinical relevance.

OTX2 is regulated during embryogenesis by several pathways including FGF- and BMP-signalling [56,57]. Accordingly, the same relation is documented in embryonal stem cells, showing that BMP-signalling inhibits and FGF-signalling activates the expression of OTX2 [58]. Here we identified aberrantly enhanced FGF2 signalling involved in the activation of OTX2 expression in the malignant context of HL. Enhanced FGF-pathway activity has been described in HL cell lines and HL patients where it correlates with poor outcome [49,50]. However, the study by Khnykin et al. [49] did not detect FGFR2 protein in HL cell lines, suggesting alternative mechanisms of FGF2 signalling. Nevertheless, OTX2 may represent a pathologically relevant downstream target of this signalling pathway in HL. In addition, we have identified aberrant activity of the WNT-signalling pathway in HL. However, this pathway did not contribute to OTX2 activation and requires more substantial investigation in this B-cell lymphoma–the HL cell line KM-H2 may represent a suitable tool for this purpose. The copy numbers of OTX1 and OTX2 are increased which may contribute to their upregulation in HL cell lines. Copy number alterations have been described in HL for several genes which accordingly show transcriptional deregulation, including gains of JAK2, REL and CYB5B and deletions of CYBB and CYLD [59–64]. Therefore, copy number variations represent a frequent chromosomal abnormality in HL. Finally, PAX5 represses OTX2 transcription in the developing brain [65]. In normal PAX5-positive B-cells this relation may keep OTX2 silent, but HRS cells frequently show reduced PAX5 expression and exhibit, thus, a decreased potential to repress OTX2 [7,8].

MSX1 is physiologically regulated by OTX2 in embryonal development of the NPBR and the brain [34,66]. Here, we identified the same connection in HL, providing yet another instance of aberrant reactivation in cancer. The transcriptional activity of OTX2 is modulated by interaction with competing cofactors: MEIS2 mediates activation and TLE4 repression of target genes [67]. Our profiling data indicate low expression levels of TL4 and high levels of MEIS2 in KM-H2 (**S4 Fig**), supporting the observed gene activating operation mode of OTX2. We were unable to detect OTX2 expression in hematopoietic cells or tissues obtained from healthy donors, including B-cells, T-cells, PBC, BM, LN and thymus, thus revealing ectopic expression in this B-cell malignancy. Deregulated expression of MSX1 and OTX2 may represent in malignant lymphoid cells the redeployment of a GRN normally restricted to the NPBR, neural crest cells and placodes [29–31]. Medulloblastomas originate from deregulated NC cells, and OTX2 is frequently overexpressed in this cancer via gene amplification, where it activates cell cycle genes and inhibits differentiation, illustrating its function as an oncogene with respect to the NPBR [68–70].

Functional analysis of OTX2 in developing retinal cells has also revealed ZHX1 to be a target gene which supports our findings of the same relation in HL [71]. Our expression data in hematopoietic cells and tissues indicate that ZHX1 is a T-cell specific gene, a finding supported by a recent analysis of a murine T-cell line [55]. The ZHX1 protein contains two Zn-fingers and five homeodomains, is destabilized via SUMOylation, and mediates gene repression by interaction with corepressors [54,72–75]. Assembling functional target genes of ZHX1 in HL may provide novel insights into the pathogenesis of this malignancy and is, therefore, an ongoing topic of our research. GATA3 activation by OTX2 is part of a conserved GRN/kernel in endo-mesoderm specification [53]. However, this ancient regulatory connection was not detected in HL cells. Taken together, the aberrant GRN identified in HL comprises OTX2, MSX1, FOXC1, PAX5 and ZHX1, resembling GRNs described in the development of the NPBR and its derivates.

The NPBR generates the NC and placodes which subsequently form major parts of the developing head including the jaw and the sensory nerves and organs. The evolutionary emergence of these fundamental cranial structures roughly coincided with lymphocytes which represent the adaptive immune system [76–80]. This situation may have abetted the coevolution of both developmental innovations by deployment of the same existing GRNs, nowadays reflected by their identical usage of several TFs in physiological development, e.g. ETS1, FOXC1, GATA2, GATA3, MYB, MYC, MSX1, RUNX1, TCF3 [29,81]. This evolutionary relation may underly matching aberrant activities of specific lymphoid malignant genes and those which physiologically regulate development of the NPBR and its derivates as documented here for OTX2.

Our study identified novel aberrantly expressed TFs in HL, namely OTX2 and ZHX1, which deregulate B-cell differentiation, thus broadening the understanding of this malignancy. Furthermore, the similarity between deregulated TFs involved in lymphoid malignancies and embryonal development of the NPBR may reflect convergent evolutionary deployment of GRNs in both lymphocytes and NC/placodes.

## Supporting Information

S1 Fig In silico expression of OTX2, OTX2-AS1 and OTX1 in primary HL, B-cell samples, and additional lymphomas (GSE12453); TCRBL: T-cell rich B-cell lymphoma, FL: follicular lymphoma, BL: Burkitt lymphoma, DLBCL: diffuse large B-cell lymphoma). (TIF)

S2 Fig Gene set enrichment analysis of top 1000 overexpressing genes in KM-H2 using DAVID. (TIF)

S3 Fig In silico expression of candidate genes in primary HL and B-cell samples and additional lymphomas (GSE12453). (TIF)

S4 Fig Heatmap of expression profiling data for TLE and MEIS genes in HL cell lines, demonstrating reduced levels of TLE4 and enhanced levels of MEIS2 in KM-H2. (TIF)

S1 Table Expression profiling data of 7 HL cell lines. (XLS)

S2 Table Top 1000 overexpressed genes. (XLS)

S3 Table Top 1000 underexpressed genes. (XLS)

S4 Table Differentially expressed genes after OTX2 knockdown in KM-H2. (XLS)
