# Supplementary figures and images for: Aberrantly Expressed OTX Homeobox Genes Deregulate B-Cell Differentiation in Hodgkin Lymphoma
Source: PLoS One. 2015 Sep 25;10(9):e0138416. doi: 10.1371/journal.pone.0138416 (PMC4583255; doi:10.1371/journal.pone.0138416)

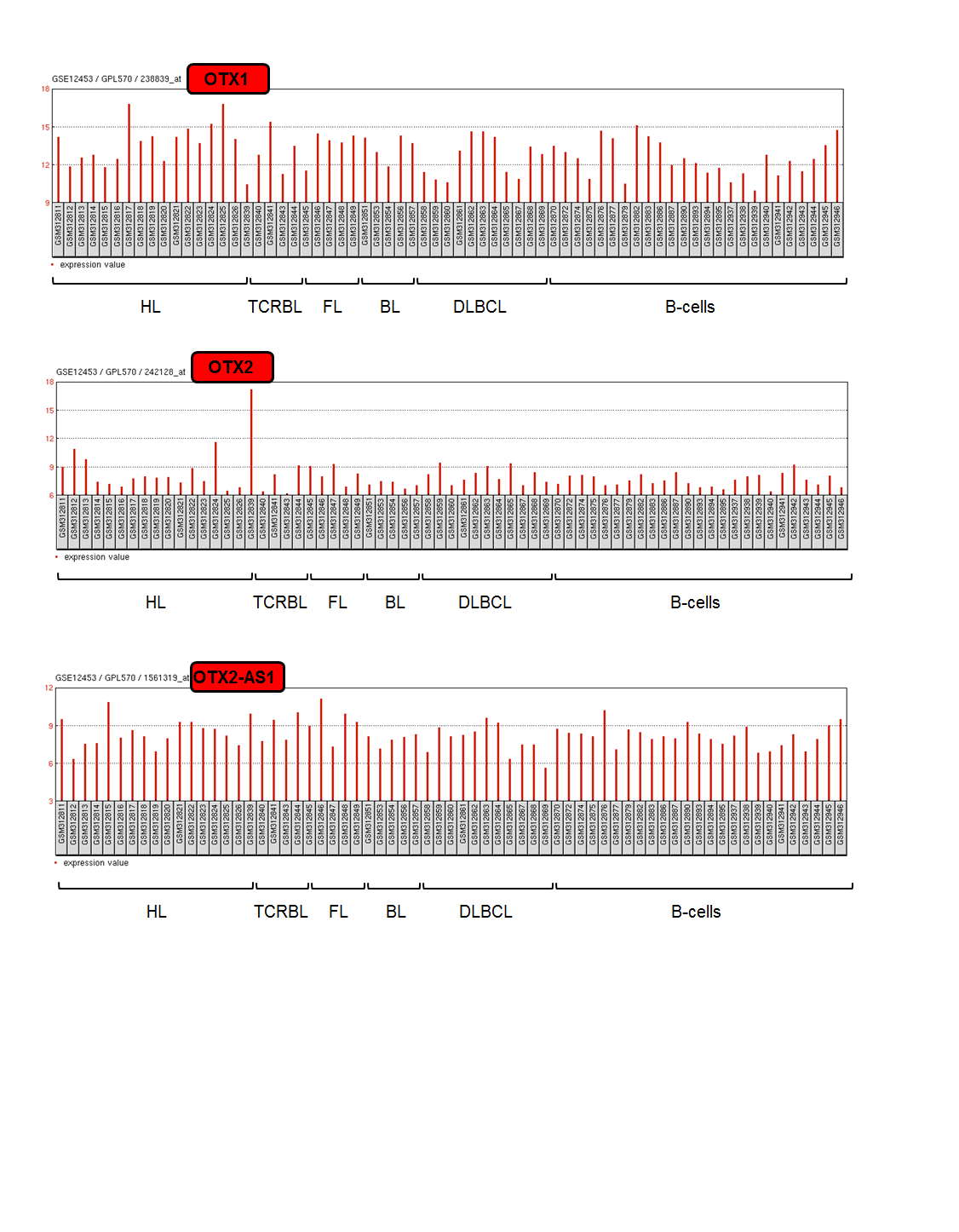

Supplement: S1 Fig — (TIF) [file pone.0138416.s001.TIF]

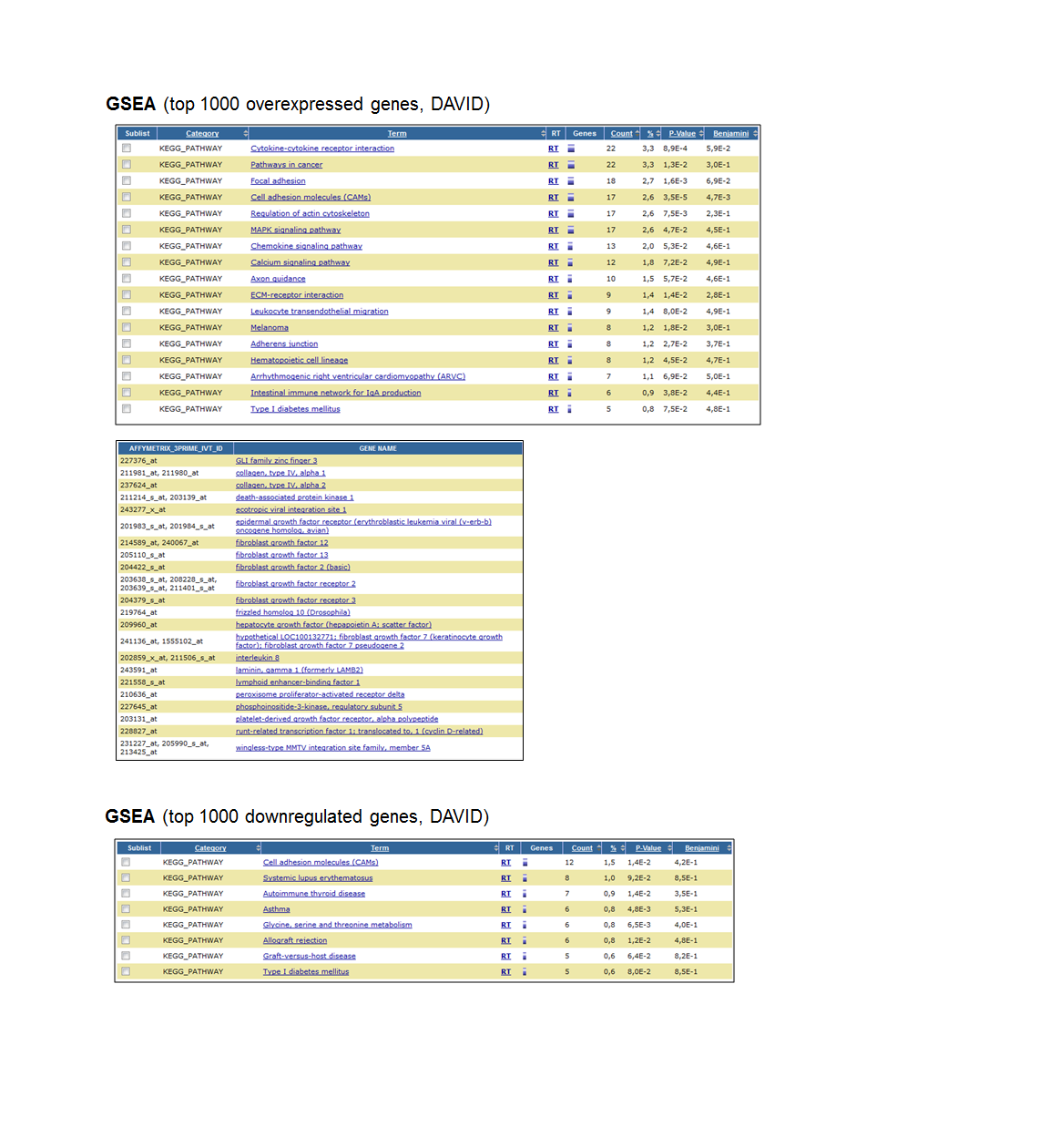

Supplement: S2 Fig — (TIF) [file pone.0138416.s002.TIF]

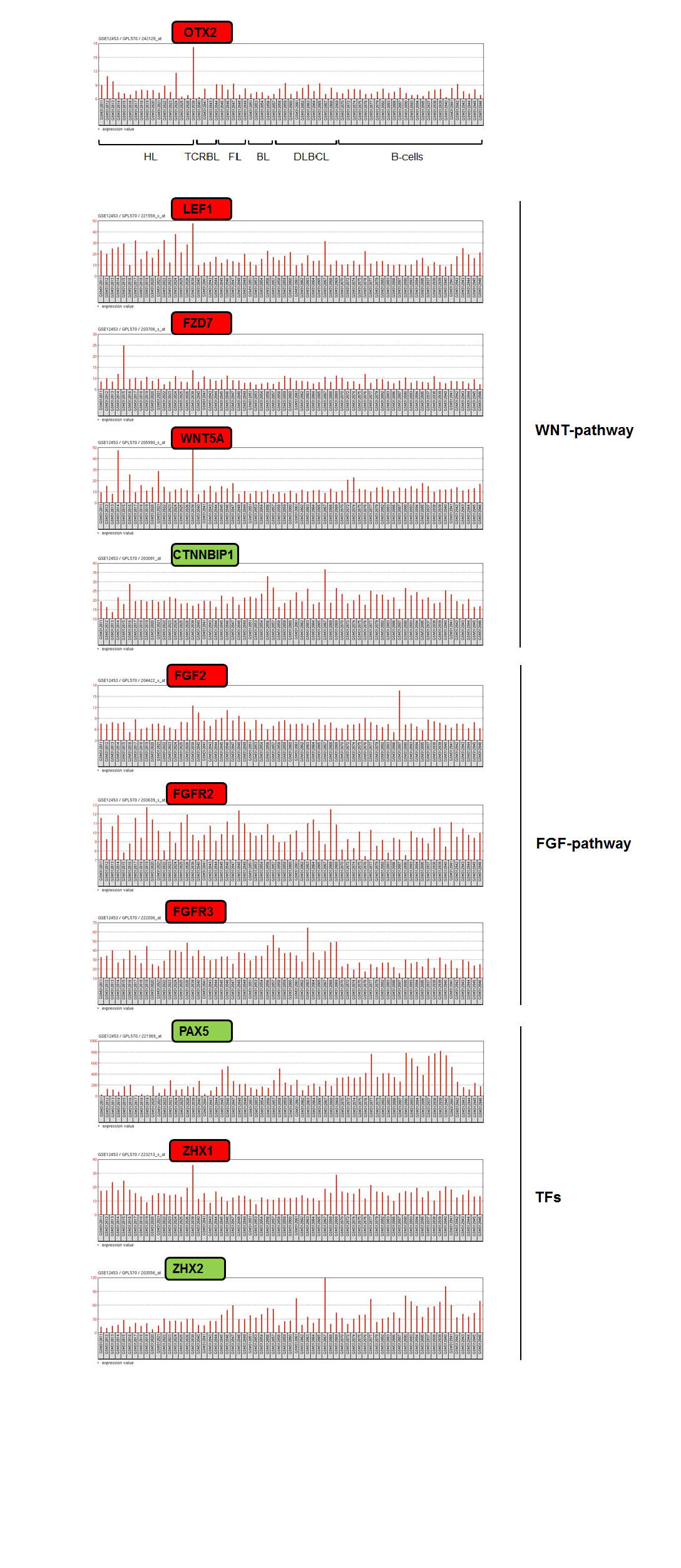

Supplement: S3 Fig — (TIF) [file pone.0138416.s003.TIF]

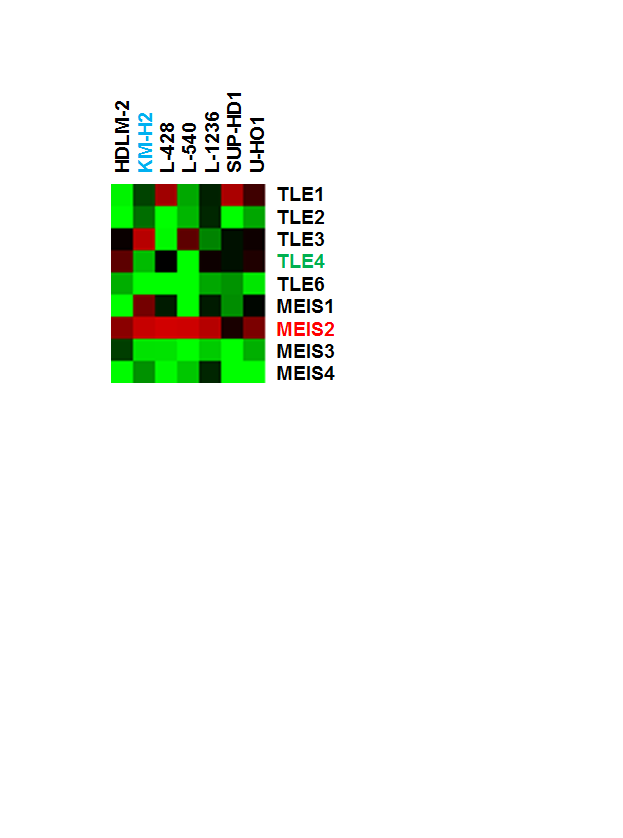

Supplement: S4 Fig — (TIF) [file pone.0138416.s004.TIF]
